# Abdominal Aortic Aneurysm Detection in Bioelectrical Impedance Cardiovascular Screenings—A Pilot Study

**DOI:** 10.3390/jcm12113726

**Published:** 2023-05-28

**Authors:** Amun G. Hofmann, Tarik Shoumariyeh, Christoph Domenig, Falko Skrabal, Johannes J. Kovarik

**Affiliations:** 1 Department of Internal Medicine III, Division of Nephrology and Dialysis, Medical University of Vienna, 1090 Vienna, Austria; 2 Department of General Surgery, Division of Vascular Surgery, Medical University of Vienna, 1090 Vienna, Austria; 3 Institute of Cardiovascular & Metabolic Medicine, 8010 Graz, Austria

**Keywords:** abdominal aortic aneurysm, impedance plethysmography, vascular surgery, medical device

## Abstract

Screening and diagnosing abdominal aortic aneurysms (AAA) are currently dependent on imaging studies such as ultrasound or computed tomography angiography. All imaging studies offer distinct advantages but also suffer from inherent limitations such as examiner dependency or ionizing radiation. Bioelectrical impedance analysis has previously been investigated with respect to its use in the detection of several cardiovascular and renal pathologies. The present pilot study assessed the feasibility of AAA detection based on bioimpedance analysis. In this single-center exploratory pilot study, measurements were conducted among three different cohorts: patients with AAA, end-stage renal disease patients without AAA, and healthy controls. The device used in the study, CombynECG, is an open-market accessible device for segmental bioelectrical impedance analysis. The data was preprocessed and used to train four different machine learning models on a randomized training sample (80% of the full dataset). Each model was then evaluated on a test set (20% of the full dataset). The total sample included 22 patients with AAA, 16 chronic kidney disease patients, and 23 healthy controls. All four models showed strong predictive performance in the test partitions. Specificity ranged from 71.4 to 100%, while sensitivity ranged from 66.7 to 100%. The best-performing model had 100% accuracy for classification when applied to the test sample. Additionally, an exploratory analysis to approximate the maximum AAA diameter was conducted. An association analysis revealed several impedance parameters that might possess predictive ability with respect to aneurysm size. AAA detection via bioelectrical impedance analysis is technically feasible and appears to be a promising technology for large-scale clinical studies and routine clinical screening assessments.

## 1. Introduction

Abdominal aortic aneurysms (AAA) are characterized by the pathological enlargement of this vessel’s diameter. While 3.0 cm is often considered the threshold for diagnosis, it has been previously reported that lower thresholds should be considered in female and Asian populations or that a diameter enlargement of 1.5-fold compared to healthy artery sections might be of more objective value [1,2]. The key risk factors for aneurysm development are smoking, hypertension, and dyslipidemia [3]. With the increasing life span of the worldwide population, the global prevalence of AAA has been increasing constantly over the last few decades [4,5]. Safe and efficient screening policies will be essential to healthcare systems in the future, and the volume of screening investigations might have to increase with changing demographics [6]. Standard screening and surveillance methods include ultrasound (US) and computed tomography (CT) [7]. Historically, US has been the method of choice for (non-ruptured) AAA diagnosis and surveillance, and large-scale studies such as the UK Small Aneurysm Trial are based on this imaging modality [1,8]. Even though US has high sensitivity and specificity, is comparably economic and simple to conduct, and poses no associated risks [9], it does suffer from limited inter-rater reliability [10] and dimensionality reduction due to its use of two-dimensional images (an issue that might be mitigated by the emergence of three-dimensional ultrasound) [11]. CT is more expensive [12], exposes patients to radiation [12,13], and, depending on the geographic location, the availability of CT scanners might be limited [14]; however, it offers accurate multislice imaging in three axes and the potential for three-dimensional reconstructions in combination with contrast enhancement [15]. Historically, the execution of contrast-enhanced computed tomography angiography (CTA) was restricted in patients with chronic kidney disease [13]. However, according to recent data, the risk of acute kidney injury might have been overstated [16]. Nevertheless, various contrast enhancers do expose patients to additional risks, including anaphylactic shock [17] or iodinated-contrast-media-induced thyroid dysfunction [18]. Alternatives to imaging for diagnosis and surveillance include serological biomarkers, and while potential candidates exist, routine clinical applicability still depends on the acquisition of robust data in future studies [19].

A different and thus far minimally explored option is based on electrical impedance, i.e., opposition to the flow of an electric current passing through the tissue. While electrograms, i.e., depictions of an electrical current, are a staple of modern diagnostics in the form of electrocardiograms (ECGs) or electroencephalograms, bioelectrical impedance analysis is traditionally limited to body composition studies. Mathematical models for the detection of aneurysms have been previously developed [20]. However, technical application highly depends on the availability of suitable devices.

Recently, bioimpedance has re-emerged as an interesting non-invasive alternative to conventional imaging techniques as it has proven to be able to be used for large-scale cardiovascular and kidney disease screenings [21,22,23] or even the prediction of arterio-venous fistula maturation [24]. Historically, the technology was additionally investigated with respect to several pathologies within different fields of clinical medicine, such as neurology, immunology, or pulmonary disease [25]. While bioelectrical impedance studies do not result in images but instead deliver quantitative outputs, the detected signals allow for inferential studies on the underlying tissues that can facilitate diagnoses and give insights regarding the volume or mass of distinct tissue compartments [26,27]. CombynECG is a certified medical device that combines a standard 12-channel ECG and bioimpedance analysis functions alongside additional features such as blood pressure and handgrip strength measurements. It has the potential to be used as a broad screening tool for cardiovascular or renal diseases while simultaneously facilitating body composition measurements [21].

In the present study, we investigated the feasibility of AAA detection via bioelectrical impedance analysis through parameters measured using CombynECG.

## 2. Materials and Methods

### 2.1. Ethics

This study was conducted in accordance with the Declaration of Helsinki and was approved by the Ethics committee of the Medical University of Vienna (Identifier: 2111/2021). All participants signed informed consent forms, and the study adhered to the good scientific practice guidelines issued by the Medical University of Vienna.

### 2.2. Study Design

The study was designed to investigate the feasibility of detecting AAA through bioimpedance signals using CombynECG. We aimed to include 60 measurements in total to adequately adjust for the hyper dimensionality problem induced by the number of parameters measured and calculated by the device (225 variables).

### 2.3. Participants

Participants were recruited at the Medical University of Vienna between June 2020 and July 2022 at the Department of Surgery, Division of Vascular Surgery and the Department of Internal Medicine III, Division of Nephrology and Dialysis. Three different groups of participants were included in the present study: patients with AAA, end-stage renal disease (ESRD) patients without AAA, and healthy controls. In total, 61 measurements were conducted. ESRD patients have anthropometric, metabolic, and cardiovascular profiles that are partially comparable to those of AAA patients, including high comorbidity rates of atherosclerosis, hypertension, and diabetes as well as altered body composition. Healthy controls and ESRD patients were combined in a mixed non-AAA group to ensure that the models would separate AAA from non-AAA individuals instead of distinguishing healthy controls from patients. Therefore, confounding through other factors that might influence bioimpedance measurements such as comorbidities or differences in skeletal muscle mass was specifically minimized.

### 2.4. CombynECG

**Figure 1:**
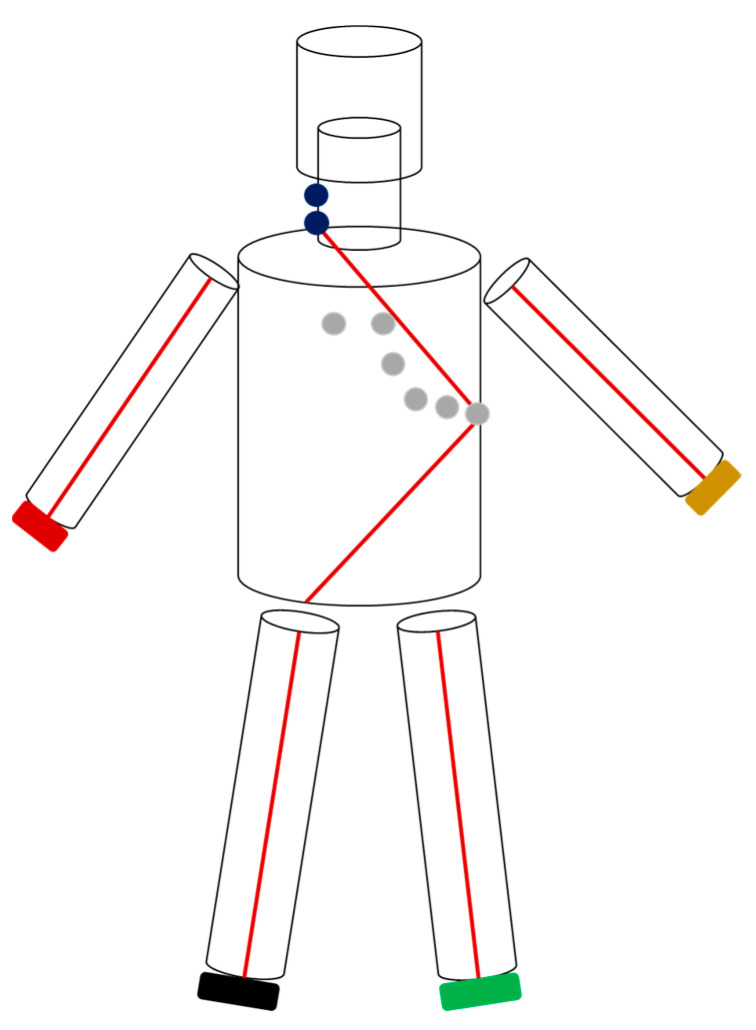
This figure illustrates electrode placement and the 6-cylinder model used by CombynECG to conduct segmental bioelectrical impedance analyses. The extremity and neck electrodes are double electrodes. Thorax parameters were derived from a signal between the proximal neck and V6 electrode, while those for the abdomen were derived from a signal from V6 to the right lower extremity.

CombynECG investigations are performed using standard 12-channel ECG electrodes placement with two additional electrodes placed on the lateral neck. Figure 1 illustrates electrode placement and signal paths. Prior to the actual measurement, the trunk, thorax, arm, and leg lengths as well as hip and waist circumference are manually measured and entered into the graphical user interface (GUI). Details of the CombynECG measurement protocol and final output have been previously described elsewhere [21,22]. In total, it takes between 10 and 15 min to run the standard CombynECG protocol. Additionally, participants’ blood pressure and handgrip strength (on the dominant side) were measured three consecutive times. While blood pressure was included in the subsequent analysis, handgrip strength was excluded. Similarly, medical history that can be entered into the CombynECG GUI was recorded but removed during pre-processing. The aim was to only include technical variables measured by the device and exclude any additional information, i.e., to establish a situation wherein the analysis is performed blind with respect to the clinical history of a patient such as their smoking history or medication intake, which could otherwise influence the algorithms by acting as confounders for the presence of AAA.

### 2.5. Analysis

All analyses were performed using R version 4.2.1 (The R Foundation, Vienna, Austria). Analyses included pre-processing raw csv output of CombynECG and subsequent model creation. For all models, an 80% training/20% test split was employed with a set seed. Feature selection was performed using Boruta [28] or through regularization via Least Absolute Shrinkage and Selection Operator (LASSO) regression. Model performance was assessed by referencing the confusion matrix of each model for its respective test set. Additionally, an exploratory association analysis was conducted to determine whether the acquired parameters could be used to approximate maximum AAA diameter. AAA diameters were manually measured in a picture-archiving and communication system (PACS) from CTA images during routine clinical work-up and extracted from written imaging reports. Although bioelectrical impedance analysis will allow for inferential estimation of volume and mass, we conducted an association analysis for the following reasons: diameters are available on written imaging study reports, which correlate very well with aneurysm volumes; an association of diameters and bioelectrical impedance signals cross-validates binary detection abilities; and treatment as well as surveillance decisions are currently based on diameters.

### 2.6. Model Development

In total, 4 different models were established to cross-validate our findings. Model 1 was constructed by removing all measurements with >10% missing values, which resulted in 51 measurements and 223 parameters. Next, variables with at least one missing data point were excluded, resulting in 51 measurements and 160 features. As a final filter, Pearson’s correlation coefficient between variables was assessed, for which 0.6 was used as a threshold for further exclusion, leaving 51 measurements and 34 variables. Boruta was used to identify important features, which resulted in 10 important parameters, which were then used to create the final model. Parameters are shown in Table 1. Finally, a random forest classifier with 500 trees and 10-fold cross-validation was built using the training dataset.

Model 2 was established not by removing any measurement or parameter a priori but via imputing missing data points on a k-nearest-neighbor graph with k = 3. Filtering based on Pearson’s correlation coefficient between variables with 0.5 used as a threshold resulted in 61 patients and 36 variables. Boruta was used to identify important features, resulting in 11 important parameters, which were then used to create the final model. Variables are shown in Table 1. In a manner analogous to model 1, a random forest classifier with 10-fold cross-validation was built from the training sample.

Model 3 was constructed by first removing all parameters with missing values, resulting in 61 measurements and 103 variables. Subsequently, Pearson’s correlation coefficient between variables was used as a filter step with 0.6 set as a threshold, resulting in 61 measurements and 20 variables. In order to investigate a different approach than that used for the first 2 models, model 3 was built around a binomial LASSO regression. Ten-fold cross-validation was performed to optimize lambda values. A minimal lambda was used for the final LASSO model built on the training sample. Non-zero penalized parameters used in the model are shown in Table 1.

Model 4 was built by first filtering variables that correlated either positively (r > 0.5) or negatively (r < −0.5) with the presence of AAA, resulting in 15 remaining variables. Subsequently, parameters that potentially reflect anatomic, hemodynamic, or body compositional changes associated with the presence of an AAA were manually selected to establish a multivariate logistic regression model in order to minimize confounding by variables that were potentially associated with pathophysiological differences between the groups independent of the AAA. Prediction on the test set was performed using a 0.5 threshold. The 5 variables fed into the model are shown in Table 1. The original variable names included in the CombynECG raw data output are shown in Supplementary Table S1.

## 3. Results

### 3.1. Proband Characteristics

Patients with AAA had a mean age of 71.1 years compared to that of 51.9 and 39.6 years for the non-AAA ESRD patients and healthy controls, respectively. The AAA group had a 27.8% proportion of women. In the non-AAA patient group, 43.8% of the measurements were conducted on female subjects, while 17.4% were conducted on healthy controls. Since the two groups were combined for the subsequent analysis, the overall rate in the non-AAA cohort was 22.4%, thereby approximately matching the AAA group. The AAA cohort had a mean maximum diameter of 5.62 cm and 3 out of 22 participants had an extensive thoraco-abdominal aneurysm. Proband information is shown in Table 2.

### 3.2. Detection of Abdominal Aortic Aneurysms

The confusion matrices for each model are presented in Table 3, Table 4, Table 5 and Table 6. Model 2 showed the best performance with 100% sensitivity and 100% specificity. Model 1 also had a sensitivity of 100% in the test portion but had a specificity of 83.3%. Model 3 resulted in a sensitivity = 66.7% and a specificity = 77.8%. Model 4, our most conservative model, had a sensitivity of 100% and a specificity of 71.4% (see Table 7). This indicates that parameters obtained from segmental bioimpedance studies can detect the presence of AAA and distinguish AAA patients from non-AAA patients and healthy controls.

### 3.3. Bioelectrical Impedance Metrics and AAA Size

Since the maximum AAA diameter is the guiding metric for surveillance and therapy decisions, all AAA patients (*n* = 22) were included in an exploratory subset analysis for this purpose. Different approaches to establishing regression models for maximum diameter were conducted, namely, fitting the intersecting parameters of models 1–4 and the disjointing set to a regular linear regression; removing variables from the original CombynECG output with missing data points; and applying a correlation filter (r = 0.5) prior to running a linear regression with stepwise forward selection. Analysis of the models showed poor performance.

**Figure 2:**
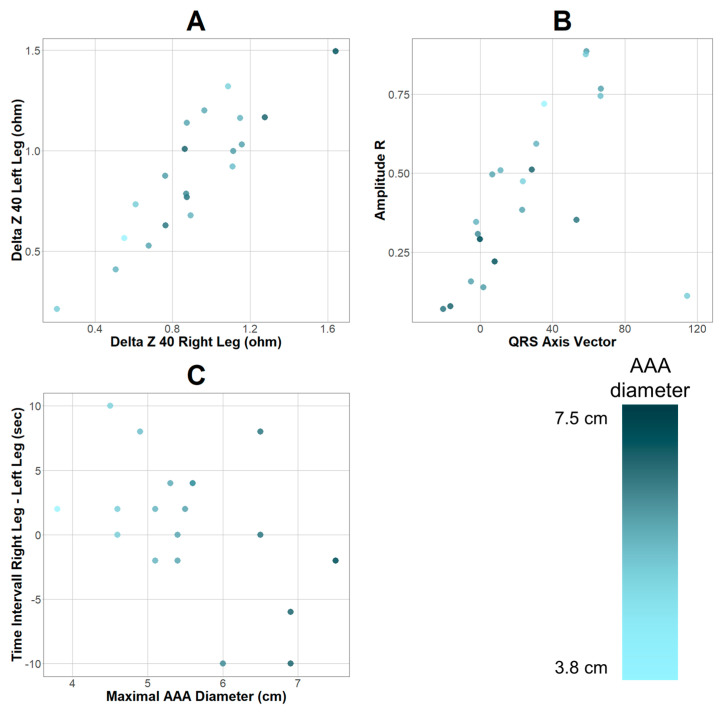
Parameters with positive (**A**) and negative (**B**) associations regarding maximum AAA diameter. (**C**) depicts the time interval between signal detection in the right and left leg plotted against AAA diameter. Gradient depending on maximum AAA diameter shown in adjacent bar. Differences between plotted points and sample (*n* = 22) are due to missing data points.

Apart from prediction, association analysis of variables and maximum AAA diameter was conducted. Filtering based on correlation coefficients of the diameter measurements taken from the CTA studies and parameters resulted in six variables based on r > 0.45 or < −0.45. (Supplement Table S2) Qualitative analysis revealed a positive association between AAA diameter and signals detected in the lower extremities (Figure 2A) as well as negative associations regarding cardiac bioimpedance variables (Figure 2B). Additionally, it was observed that with an increasing AAA diameter, the variance of the time interval between signal detection in the lower extremities simultaneously increased (Figure 2C).

## 4. Discussion

In this study, segmental bioimpedance measurements combined with a standard 12-channel ECG were investigated for their ability to detect AAA in a mixed study population consisting of patients both with and without AAA as well as healthy controls. Due to the hyper-dimensionality problem caused by the 225 output variables and the limited sample size in this pilot study, feature selection was performed, and four different machine learning models were subsequently established. Model 1, a random forest algorithm applied to a filtered dataset and with selected predictors based on Boruta, showed satisfying performance in its ability to detect AAAs, achieving no false negatives and one false positive in the test partition. Model 2 was the best-performing model, achieving 100% accuracy in the test sample. Model 3, a LASSO regression performed on a filtered dataset, had the worst performance, with a specificity of 77.8% and a sensitivity of 66.7%. Model 4, the most conservative model, also showed reliable performance regarding the evaluation of a potential screening tool, presenting 100% sensitivity in the test set. While solid regression models for approximating maximum AAA diameter could not be established, an association analysis yielded promising candidates for further exploration in larger cohorts such as the variance in the time interval of signal detection in the lower extremities.

The potential application of bioimpedance analysis for the detection of aneurysms has been previously discussed and repeatedly been praised as a non-invasive, low-cost screening technology [20,29,30]. Additionally, changes in body composition have recently been shown to be associated with abdominal aortic aneurysm growth after endovascular treatment [31]. CombynECG uses segmental bioimpedance including a routine ECG that can also reliably assess body composition [21,22] and grants insight into hemodynamic parameters such as the aortofemoral pulse wave velocity [32]. Based on these premises, it appears very reasonable that the device can facilitate AAA diagnosis and provide a non-invasive screening tool.

In the present test partitions of limited sizes, adequate levels regarding sensitivity and specificity were reached to allow for the establishment of sufficient evidence for follow-up investigations. However, considering the high reported sensitivity (94–100%) and specificity (98–100%) of ultrasound investigations for AAA detection [9], it is reasonable to require similar thresholds, e.g., >90%, for bioelectrical-impedance-based studies before this technology can be introduced in clinical practice for this specific application. Not all investigated models could fulfill these criteria in this study. Nevertheless, the used medical device is not specifically designed for AAA diagnostics, and missing data points might have impaired the overall accuracy of the investigation. Some data were missing due to technical issues during the measurement protocol, which is a common problem faced in the introduction of new devices and their associated learning curves. Additionally, with the potential to specifically design next-generation bioelectrical-impedance-based devices to facilitate AAA detection from an engineering perspective, it seems reasonable to assume that the technology has not yet reached its performance peak for the investigated application.

The required training sample size for the application of machine learning algorithms is currently being debated and is a matter of great interest in clinical research [33]. The sample size in the present study certainly constitutes a limitation and is prone to introducing bias during model development and might inflate accuracy in the test partitions [34]. Nevertheless, even the conservative conventional logistic regression, model 4, had good accuracy during prediction. Therefore, the performance of the more complex machine learning models is unlikely to be driven by bias, confounding, or chance alone. The identified predictor variables and derived models might not constitute the best possible options but provide sufficient evidence for technical feasibility.

Technical issues during the execution of CombynECG measurements, such as the temporary loosening of an adhesive electrode, were encountered that led to missing data points. A filtering workflow that included removing variables with missing data points, for example, led to the loss of more than 50% of all possible predictors. However, these errors are at least partially attributed to the learning curve associated with the use of new diagnostic devices. Furthermore, even the loss of possible predictors resulted in acceptable performance, as evidenced with model 3, and even our most conservative approach in the present project, model 4, had a sensitivity of 100%. The best-performing model was established by statistically imputing missing data points on a k-nearest neighbor graph, thus creating an inherent bias. However, all four models showed predictive ability, thereby cross-validating our main finding: AAA detection via bioimpedance analysis is not only theoretically possible but is achievable with open-market-accessible devices.

In the present work, we present a potential alternative to image-based AAA screenings. Similar to ultrasound studies, segmental bioelectrical impedance analysis is radiation- and contrast-enhancement-free and excludes the inter-rater variation of sonographic studies. While the benefit of imaging studies is the provided information on anatomic features, bioelectrical impedance analysis allows for the simultaneous assessment of other physiological features and pathologies including body composition [21], heart failure [22,23], and chronic kidney disease [23,25], rendering the technology an interesting candidate for cardiovascular screenings. Additionally, such analyses are cost-effective to conduct and do not require execution by a radiologist or specialist since they can be facilitated by all professions permitted to conduct ECG studies. Segmental bioelectrical impedance analysis should not be perceived as a competitor to imaging studies for AAA detection but as a further option to lower the threshold for populational screening efforts.

Ultimately, promising results were discovered with a device that was not specifically designed for the research question at hand. While residual confounding as a limitation of our data analysis certainly persists and analyses were conducted in a limited sample size, the level of evidence legitimizes further exploration in larger trials. Additionally, the present investigation could lead to spin-off studies regarding aneurysms in other anatomic locations such as the thoracic aorta or popliteal artery. With the renaissance of bioimpedance in the field of diagnostics, future devices could be engineered to optimize their potential for AAA detection and diameter or even volume approximation, for example, through the application of additional abdominal electrodes. Additionally, signals from bioimpedance studies can theoretically be translated into images, even though this process is currently limited to investigational applications [35]. However, further research on electrical impedance tomography has the potential to precipitate disruptive changes. While this technique cannot supplant imaging modalities for the planning of open or endovascular treatments currently, it might be able to facilitate simple and cost-effective screenings and subsequent surveillance.

## Figures and Tables

**Table 1:** CombynECG parameters used for the construction of each model after feature selection. (lf = low frequency; lf/hf = low frequency/high frequency ratio; ECF = extracellular fluid; and ICF = intracellular fluid.)

	**Selected Parameters**		
Model 1	Amplitude T Q to 1st Heart Sound Reactance (40 kHz)—Left Leg Vector QRS Axis	Volume Wave Velocity Mean Hip Circumference PQ Time	Power Spectral Density (lf) Body Fat Max. Amplitude—Thorax z40
Model 2	Peak Time Interval Thorax—Left Arm Thorax Length PQ Time Trunk Length	Beat–Beat Stroke Index Mean Amplitude Q ECF/ICF Ratio—Abdomen 1st/2nd Hear Sound Ratio	Waist/Hip ratio Q to 1st Heart Sound Time Interval Q—Beginning dZ/dt
Model 3	Thorax Length Hip Circumference Waist/Hip Ratio Steepness Increase dZ Thorax	Steepness Increase—Left Leg Steepness Decrease—Right Arm Q to 1st Heart Sound	Power Spectral Density (lf/hf) Impedance (400 kHz)—Left Arm Impedance (400 kHz)—Thorax V6
Model 4	Waist Circumference Volume Wave Velocity	Hip Circumference ECF/ICF Ratio—Abdomen	Q to 1st Heart Sound

**Table 2:** Characteristics of the study population. Age is given as arithmetic mean (standard deviation), BMI is depicted as median (Q1–Q3), blood pressure is given as systolic/diastolic mean of three measurements (2 missing in healthy controls and 2 in ESRD patients), and AAA diameter is given as group mean ± standard deviation. AAA = abdominal aortic aneurysm, ESRD = end-stage renal disease, and TAAA = thoraco-abdominal aortic aneurysm.

	Patients with AAA	ESRD Patients *w*/*o* AAA	Healthy Controls
N	22	16	23
Male/female	16:6	9:7	19:4
Age (years)	71.1 (8.2)	51.9 (17.4)	39.6 (13.6)
BMI (kg/m^2^)	27.7 (25.4–30.9)	22.2 (19.4–25.3)	22.6 (20.5–24.6)
Blood pressure (mmHg)	144/82	146/84	132/75
Antihypertensive treatment	22	16	1
AAA diameter in CTA (cm)	5.6 ± 1.0	-	-
AAA:TAAA	19:3	-	-

**Table 3:** Model 1 confusion matrix.

		Prediction	
Reference		Aneurysm	No aneurysm
	Aneurysm	4	0
	No aneurysm	1	5

**Table 4:** Model 2 confusion matrix.

		Prediction	
Reference		Aneurysm	No aneurysm
	Aneurysm	5	0
	No aneurysm	0	7

**Table 5:** Model 3 confusion matrix.

		Prediction	
Reference		Aneurysm	No aneurysm
	Aneurysm	4	2
	No aneurysm	2	7

**Table 6:** Model 4 confusion matrix.

		Prediction	
Reference		Aneurysm	No aneurysm
	Aneurysm	5	0
	No aneurysm	2	6

**Table 7:** Performance of each model regarding the detection of AAA.

	Specificity	Sensitivity
Model 1	83.3%	100%
Model 2	100%	100%
Model 3	77.8%	66.7%
Model 4	71.4%	100%

## Data Availability

Data access can be granted upon reasonable request to the corresponding author.

## Supplementary Materials

The following supporting information can be downloaded at: https://www.mdpi.com/article/10.3390/jcm12113726/s1, Supplementary Table S1. CombynECG parameters defined in the raw output file used for the construction of each model after feature selection. Supplementary Table S2. CombynECG features with correlation coefficient < −0.6 or >0.6 regarding maximal AAA diameter.
