# Supplementary material for: Abdominal Aortic Aneurysm Detection in Bioelectrical Impedance Cardiovascular Screenings—A Pilot Study
Source: J Clin Med. 2023 May 28;12(11):3726. doi: 10.3390/jcm12113726 (PMC10253797; doi:10.3390/jcm12113726)
Supplement: Supplementary file 1 [file jcm-12-03726-s001.zip › jcm-2343106-supplementary.pdf]

Supplement Table S1. CombynECG parameters as defined in the raw output file used for the construction of each model after feature selection.

	Selected parameters		
Model 1	amp_T_mv<br>t_Q_S1_msec<br>imp_p_LL400<br>qrs_axis_deg	t_VW_mean_ms_z<br>Hip<br>t_PQ_msec	psepc_lf_ms2<br>fat_kg_z<br>amp_max_TH_z40
Model 2	T_TH_LA_msec<br>b2b_si_mean<br>Len_Thorax<br>t_PQ_msec	amp_Q_mv<br>Len_Trunk<br>ecf_icf_ABD<br>S1_S1S2_ratio	WaistHip<br>t_Q_S1_msec<br>t_Q_startslope_msec
Model 3	Len_Thorax<br>Hip<br>WaistHip<br>sl_TH	sl_LL<br>fl_RA<br>t_Q_S1_msec	pspec_lfhf<br>imp_LA400<br>imp_TH400_v6
Model 4	Waist<br>t_VW_ms	Hip<br>ecf_icf_ABD	t_Q_S1_msec

Supplement Table S2. CombynECG features with correlation coefficient < -.6 or > .6 regarding maximal AAA diameter.

Parameter	**Correlation coefficient**
amp_AC_RL_ohm	**.53**
amp_max_RL_ohm	**.47**
-	-
qrs_axis_deg	**-.45**
area_LA_ohm	**-.46**
amp_R_mv	**-.47**
t_RL_LL_msec	**-.47**
